# Initial oxidation behavior of a single crystal superalloy during stress at 1150 °C

**DOI:** 10.1038/s41598-020-59968-3

**Published:** 2020-02-20

**Authors:** Jinyao Ma, Wenxiang Jiang, Jin Wang, Yuefei Zhang, Ze Zhang

**Affiliations:** 1 Institute of Microstructure and Property of Advanced Materials, Beijing University of Technology, Beijing, 100124 China; 2 School of Materials Science and Engineering, Zhejiang University, Hangzhou, 310058 China

**Keywords:** Engineering, Materials science

## Abstract

Revealing the initial oxidation behavior of single crystal superalloys is significant for a better understanding of the oxidation mechanism of turbine blades during service condition. The purpose of current research was to observe the initial oxidation of a single crystal superalloy. *In-situ* oxidation experiment during only thermal exposure and thermal-stress pattern were carried out. The mechanism of nucleation and growth of oxide scale was discussed. Results showed that the oxide on the interface of γ/γ′ phase was constituted of Al_2_O_3_ precipitates and formed by external diffusion of Al atoms or ions. Loading stress enhanced the diffusion of Al atom causing high oxidation rate. A logarithmic model was proposed and fitted well with the oxidation process.

## Introduction

Nickel-based single crystal superalloys have been widely used in turbine blades and other hot-end components of modern aeroengines due to their excellent creep and mechanical properties^1,2^. To improve turbine efficiency, increasing turbine entry temperature is challenging superalloy’s operational limits^3^. Such high temperature also causes the rotating components (turbine discs and blades) to bear high centrifugal stresses^4^. Actually, except for high temperature and heavily stress, oxidation and hot corrosion unavoidably degrade the performance of turbine blades during service condition^5,6^. Though thermal barrier coating (TBC) technology has been developed to avoid direct contact between high-temperature gas and turbine blades^7^ and also prevent the direct contact between oxygen and blades to some degree, the oxidation is still inevitable especially when the spallation or cracking of the TBC occurs. In many cases oxidation has been described as the precursor to fatigue damage^8^ and cause of inferior creep performance of thin wall superalloy^9^. Hence for better understanding the oxidation process is of great importance.

Though there are extensive researches focusing on oxidation behavior of superalloy during intermediate and high temperature^10–12^, the initial oxidation process has not been observed clearly considering the rapid formation of adherent oxide scale. Through *in-situ* environmental transmission electron microscopy (ETEM), initial oxidation at only thermal exposure was observed by Ding^13^, and gave the proof that oxygen diffusion path is more inclined to be the interface rather than matrix channels^14^. However, considering the real service condition, the temperature of turbine blades can reach about 1150 °C even 1200 °C during emergency regimes^15^, and the loadings in [001] direction caused by thermal stress vary by around 400 MPa^16^. Researches of initial oxidation combining both thermal and stress are rarely reported until now.

In this paper, microstructure evolution during oxidation of a nickel-based single crystal superalloy during only thermal exposure and thermal-stress pattern was studied by carrying out *in-situ* experiments in scanning electron microscope (SEM) at 1150 °C and 1150 °C/330 MPa under an oxygen partial pressure of 2 × 10^−9^ atm. The oxide scale was characterized by transmission electron microscopy (TEM) qualitatively. The mechanism of oxide nucleation and growth was discussed.

## Methods

### Materials

**Figure 1:**
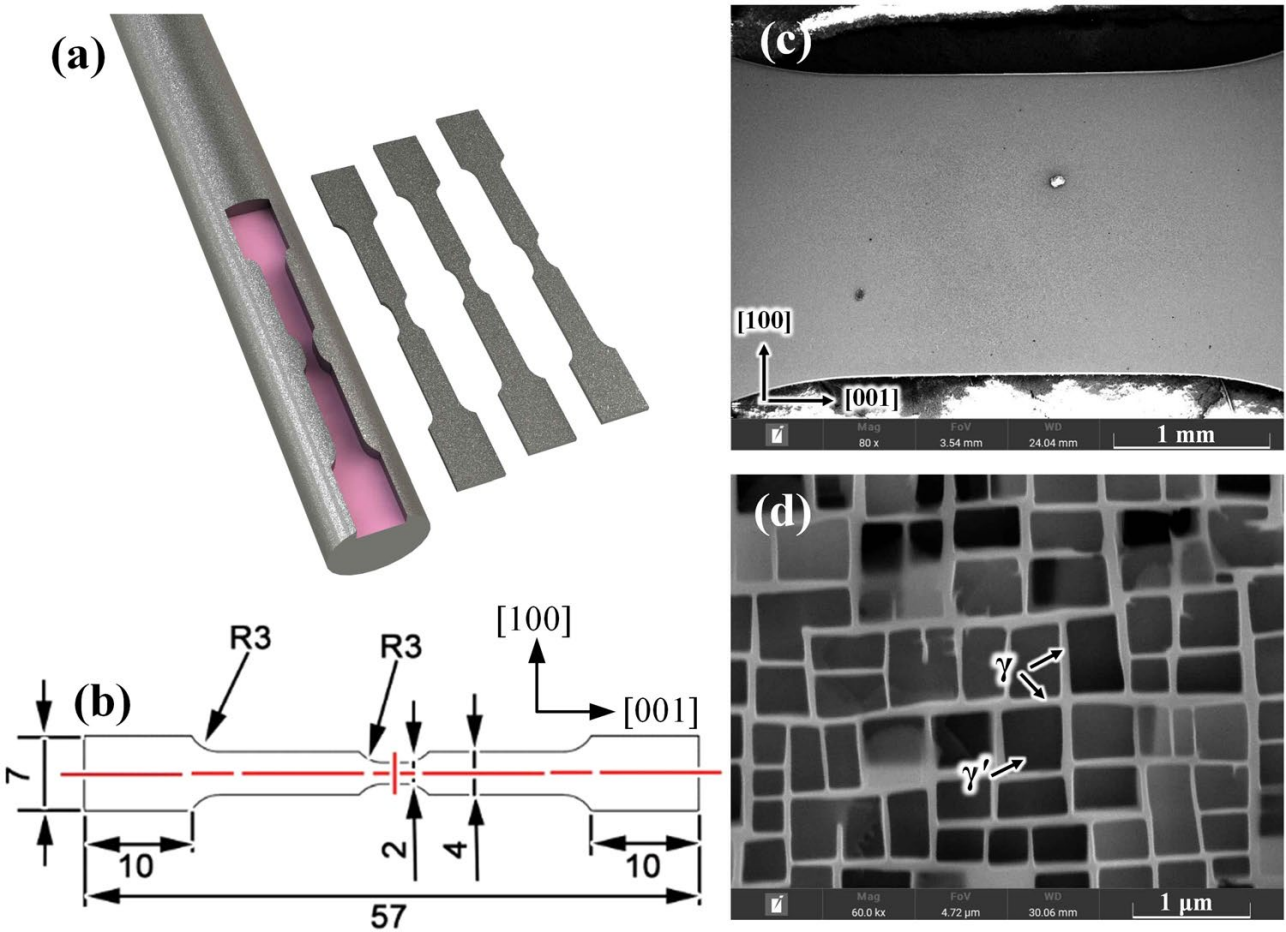
*In-situ* heating tensile specimen. (**a**) shape, (**b**) dimension, (**c**) micrograph of low-magnification and (**d**) micrograph of high-magnification.

The [001] orientation nickel-based single crystal superalloy bar is 170 mm in length and 15 mm in diameter and its chemical composition are 4.3 wt% Cr, 9.1 wt% Co, 8.0 wt% W, 5.2 wt% Al, 6.0 wt% Ta, 2.5 wt% Re, 1.5 wt% Mo, 0.1 wt% Hf, 0.5 wt% Nb and others is Ni. Standard heat treatment is performed referring to the previous articles^17,18^. Then, the alloy bar was processed into *in-situ* test specimens along [001] direction and perpendicular to [010] direction, the shape of specimen is shown as Fig. 1(a) and the dimension is marked as Fig. 1(b). The narrow region in the middle of the specimen is used for microstructure observation in SEM. The specimen is mechanically polished up to a mirror, then etched in a solution made of HNO_3_(40%)+ H_3_PO_4_(12%)+ H_2_SO_4_(48%) for 10 seconds. Figure 1(c) shows the specimen global micrograph in low magnification, and γ microstructure is illustrated in Fig. 1(d). it shows that the γ′ cuboids (ordered L12 phase, average cube edge length: 450 nm) are separated by thin brighter γ channels (fcc crystal structure, average channel width: 80 nm) and distribute uniformly all over the material.

### Instrument

**Figure 2:**
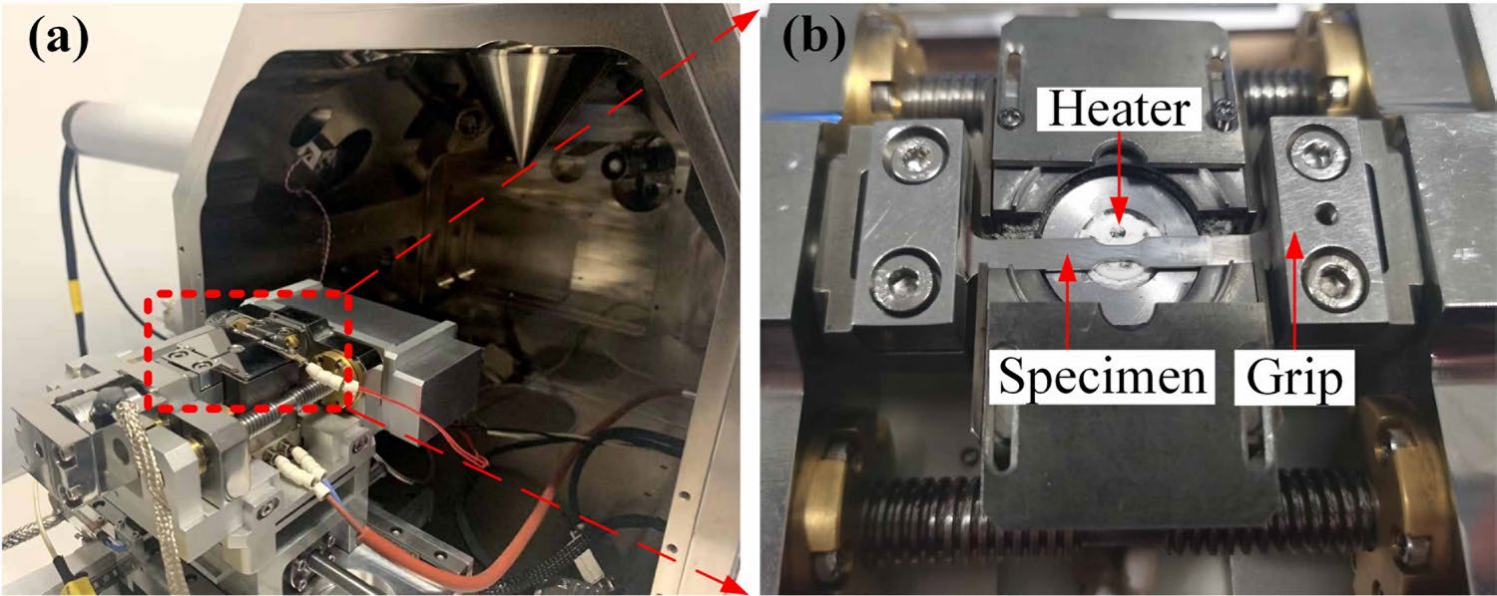
(**a**) *In-situ* tensile test equipment, (**b**) High-temperature tensile test machine.

*In-situ* tensile test equipment developed by coauthors consists of the high-temperature tensile test machine shown as Fig. 2(b) and SEM (TESCAN S8000) shown as Fig. 2(a). The nickel-based single crystal superalloy specimen is fixed on a loading frame grip and is right above the heater’s surface shown as Fig. 2(b). The heating area is 8 mm in diameter at center of the specimen and a thermocouple contacts the back surface of specimen. *In-situ* experiment under thermal exposure at 1150 °C and thermal exposure with 330 MPa was carried out in the SEM vacuum chamber, chamber pressure is kept at 10^−3^ Pa, the oxygen partial pressure is about 2 × 10^−9^ atm, the real-time observation was conducted during the experiment.

Element distribution before and after the experiment was characterized in FEI Titan G2 TEM by OXFORD X-Max energy dispersive spectrum (EDS), samples were extracted by FEI Helios NanoLab FIB from the specimen.

### The pattern of heating and stress loaded

**Figure 3:**
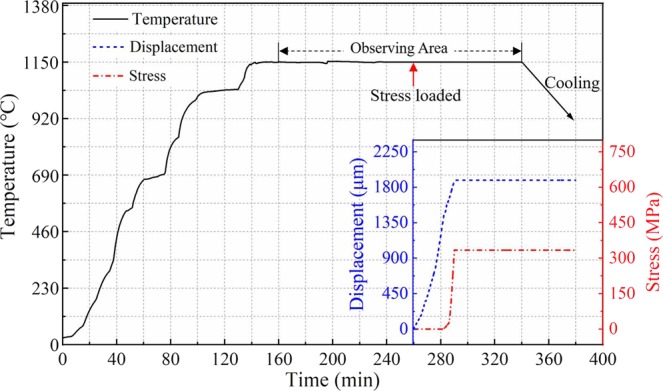
The pattern of heating process and stress loaded during experiment.

In this paper, the heating process was realized through electrical heating by adjusting input voltage in a range of 0 ~ 8.3 V, and the stress loaded process was achieved by uniaxial tensile, the details of equipment has been reported in our previous study^19^, Fig. 3 shows the temperature, displacement, and loading stress at different time during the experiment. In the beginning, temperature increased by increasing the voltage, turning points in temperature curve is caused by a sudden increase in voltage. The temperature reached 1150 °C after heating for 140 min. Then, it kept at 1150 °C and this period could be called thermal exposure. After thermal exposure for 100 min, Stress was loaded with a tensile displacement which was 1 μm/s, shown as the red and blue dashed lines in Fig. 3. Although a force about 50 N was preloaded before heating, the stress didn’t increase immediately with stress loading process considering the effect of thermal expansion. When the stress reached 330 MPa, the displacement was quitted to keep stress stable. *In-situ* observing time was located in the rage of 160 ~ 340 min. In the end, the specimen was cooled in furnace to room temperature, and stress was unloaded before specimen was taken out.

## Result

### Microstructure evolution

**Figure 4:**
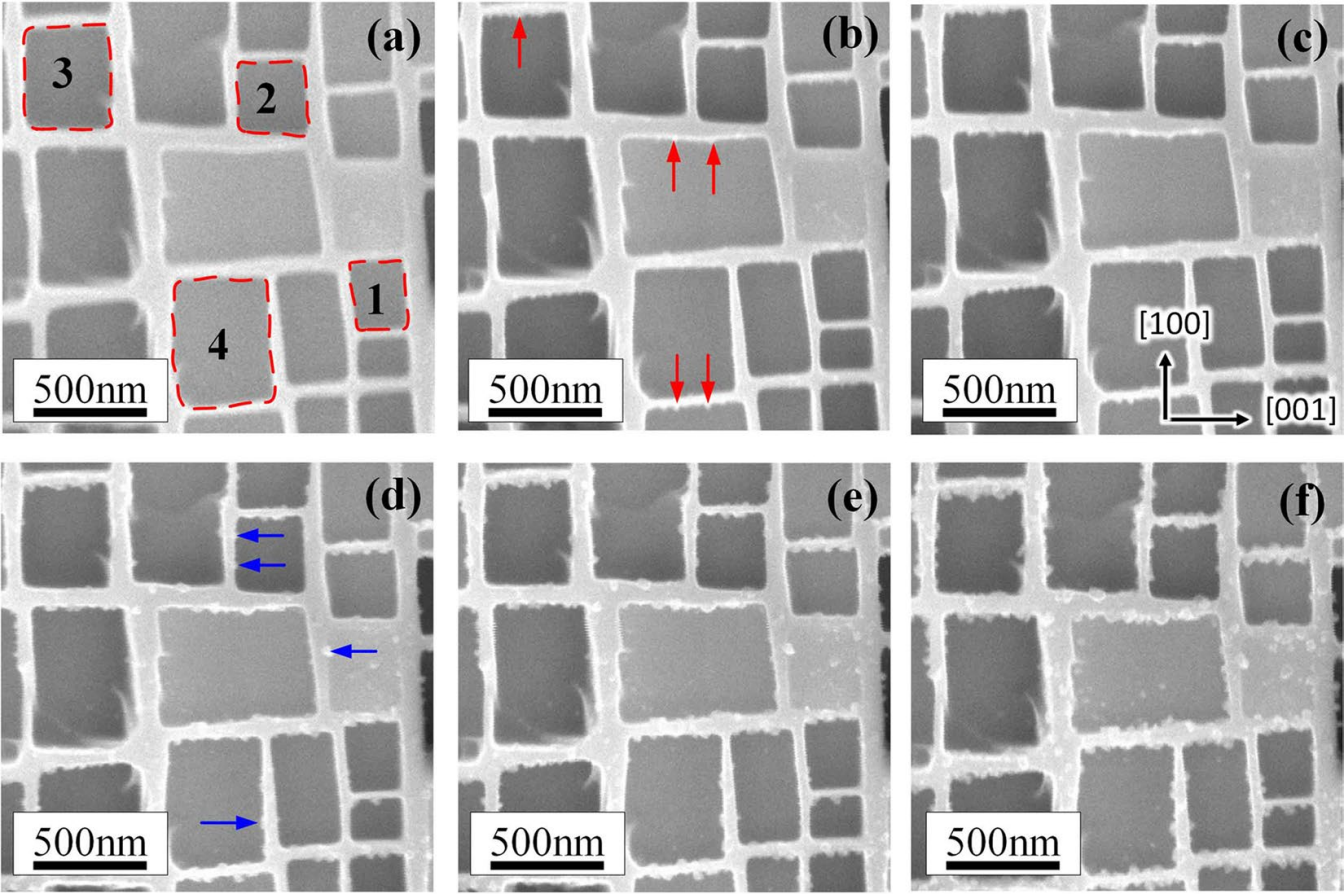
SEM images showing the microstructure evolution at 1150 °C during thermal exposure for (**a**) 20 min, (**b**) 40 min, (**c**) 50 min, (**d**) 80 min, (**e**) 100 min and (**f**) 120 min.

Figure 4 shows a series of microstructure of superalloy during 1150 °C thermal exposure condition for different times. After 20 min of thermal exposure, the microstructure of specimen is the same as the initial one. Small precipitates appear on the origin interface of γ′/γ phase after thermal exposure for 40 min and prefer gathering along [001] direction, as indicated by red arrows in Fig. 4(c). After specimen oxidized for 80 min, the small precipitates also emerge and locate along [100] direction, shown by blue arrows in Fig. 4(d). With thermal exposure time increasing, the precipitates grow slowly on the interface. Also, the precipitates on γ′ phase show the trend of nucleation and growth, clearly shown in Fig. 4(e,f).

**Figure 5:**
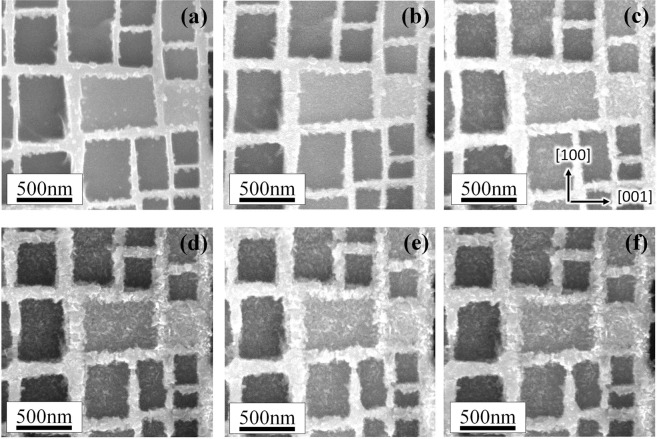
SEM images showing the microstructure evolution with stress loaded during thermal exposure at 1150 °C for (**a**) 120 min, (**b**) 135 min, (**c**) 150 min, (**d**) 175 min, (**e**) 190 min and (**f**) 200 min.

After thermal exposure oxidation for 120 min, stress was loaded to the same specimen along [001] direction and increased gradually to 330 MPa. Although the stress dose not increase immediately, the displacement (or more precisely of the strain) of specimen has increased with time, as displayed in Fig. 3. It appears the precipitates grow more rapidly while the stress was loaded. Precipitates are well advanced after 135 min thermal exposure and 15 min stress loading, they have fully occupied the original interface, as shown in Fig. 5(b). The growth of precipitates observed in Fig. 5(c) is more obvious, and it shows the direction of precipitate’s growth is outward, which makes the area marked as dashed lines in Fig. 4(a) become small. Then, the growth of precipitates tends to be slow after 175 min, shown as Fig. 5(d–f).

### Element distribution

**Figure 6:**
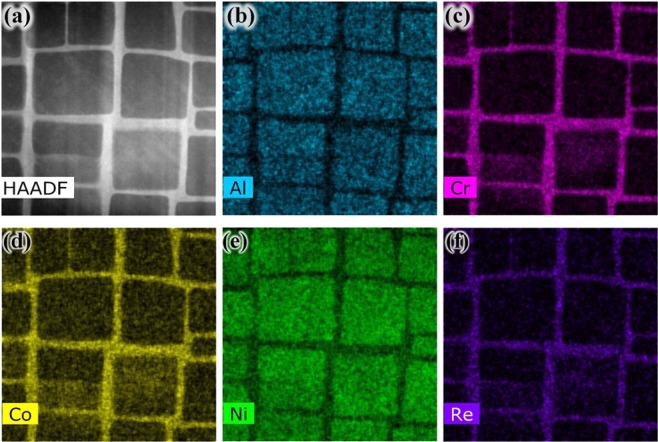
(**a**) HAADF STEM image of surface of the specimen before oxidation, (**b**–**f**) EDS element distribution for Al, Cr, Co, Ni and Re on the surface of specimen before oxidation.

Figure 6(a) presents a HAADF STEM overview image of surface of the specimen before the experiment, typical and cuboidal γ/γ′ microstructure can be seen clearly, and the interface of them is obvious. Figure 6(b–f) show a representative element distribution maps of the specimen characterized by EDS, the Ni_3_Al γ′ phase displays concentrations of Al and Ni. The γ phase mainly contains Ni (at lower level compared with γ′ phase), Cr, Co and Re, those elements are separated in γ channels uniformly.

**Figure 7:**
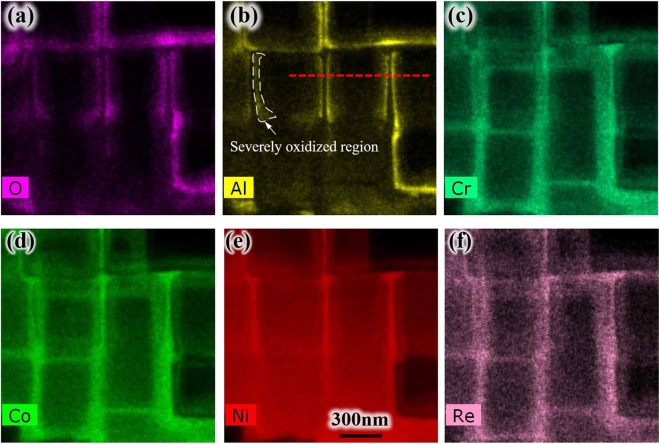
(**a**–**f**) EDS element distribution for O, Al, Cr, Co, Ni and Re on the surface of specimen after oxidation.

After the oxidation experiment, the element distribution of air-cooling specimen was mapped in Fig. 7(a–f) by EDS, Al and O elements show almost the same distribution, it indicates the precipitate are mainly Al_2_O_3_, which is also found in γ channel reported by Weiser^20^. In this paper, more precisely, nucleation on the original interface of the two phases or called the side surface of γ phase was clearly observed. The diffusion toward γ′ phase of elements Cr, Co and Re occur slightly. While the γ phase is covered with an Al_2_O_3_ oxide layer, Al is being depleted next to the oxide-alloy interface. Then Ni becomes enriched at Al depleted zone shown in Fig. 4(e), and this phenomenon is analogous to Wagner’s experiments on Cu-Pt and Cu-Pd alloys^21^. Actually, the original interface of γ/γ′ phase, side surface of γ phase and oxide-alloy interface is the same interface.

**Figure 8:**
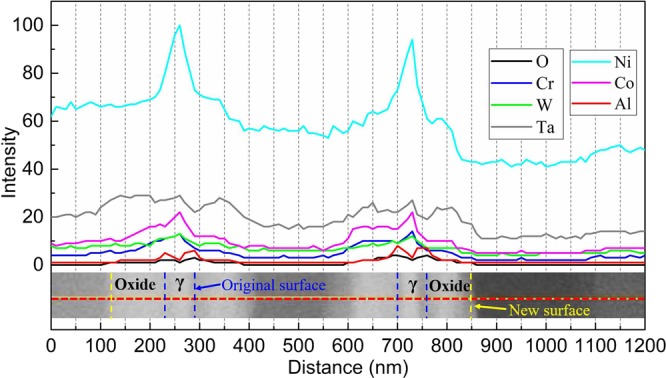
EDS line scans taken across the surface of specimen after oxidation experiment.

In order to quantify the composition at different positions after oxidation, several EDS line scans were taken from the surface of specimen and crossed both γ and γ′ phases, which is shown by the horizontal red dashed line in both Fig. 7(b) and Fig. 5. The alloy-oxide surface is marked as the blue dashed lines in Fig. 8 according to the high content of Al and O elements caused by diffusion and adsorption, respectively. The width of γ channel is about 70 nm close to the average width 80 nm described in Fig. 1(d). Moreover, Ta, W, Co, Cr, and even Re show slightly diffusion in oxide scale, which is caused by concentration gradient. Also, some compound oxide might form nearby new surface of oxide scale, but it could be negligible. The new surface is displayed by the yellow dashed lines in Fig. 8. It can be seen the thickness of oxide scale in sideway direction could reach almost 90 nm. This value was little higher than that analyzed in discussion section since the oxidation was more likely proceeding during the cooling period and also different zone was selected.

**Figure 9:**
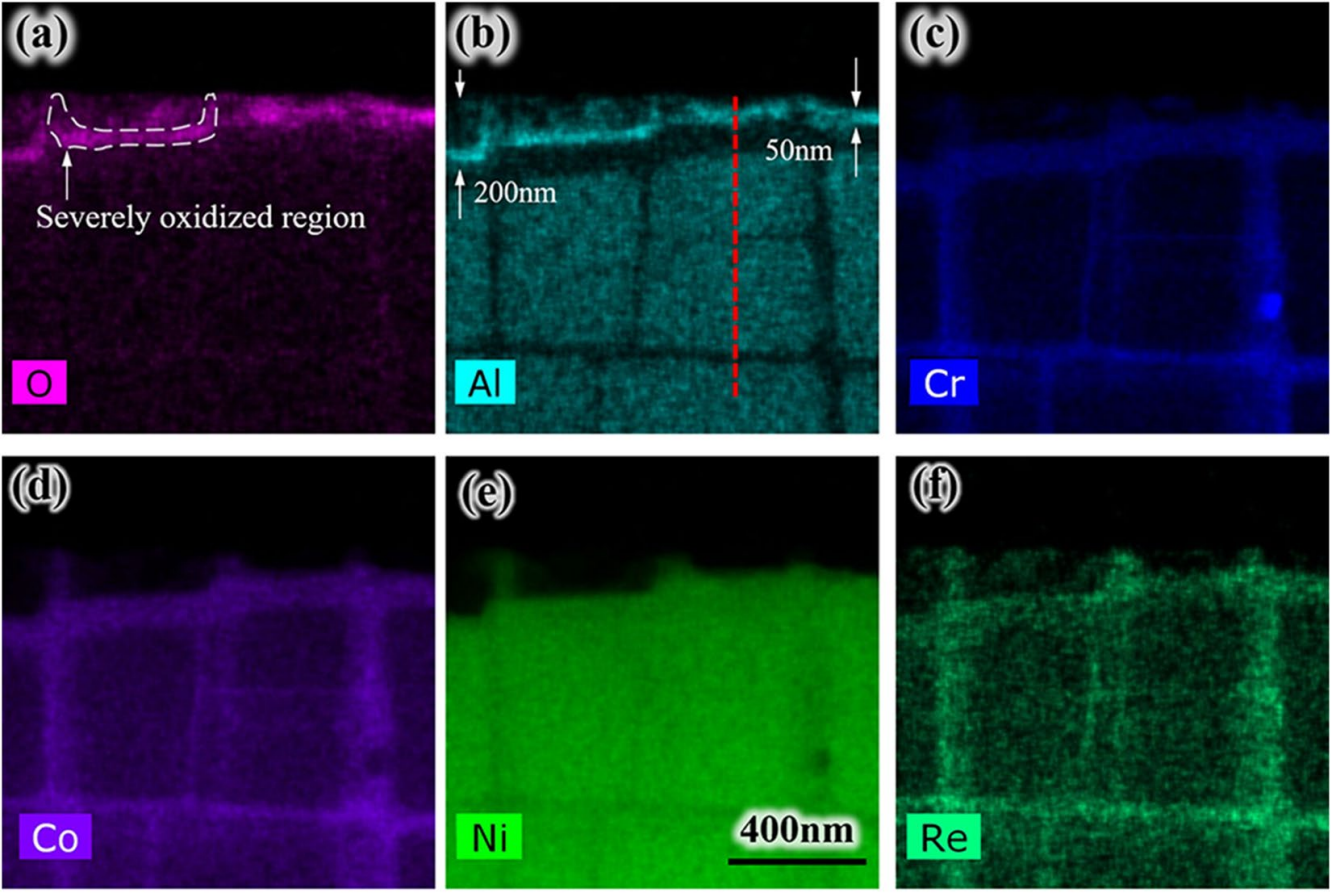
(**a**–**f**) EDS element distribution for O, Al, Cr, Co, Ni and Re in depth direction of specimen after oxidation.

Figure 9(a–f) shows the element distribution for O, Al, Cr, Co, Ni and Re in depth direction of specimen after oxidation. It can be seen on the bottom interface γ/γ′ phase also occurred oxidation. And the severely oxidized zone circled by the white dashed line in Fig. 9(a) analogies that in Fig. 7(b), which indicates the oxidation occurred on both side and bottom interfaces of γ/γ′ phase. It also could be deduced from the change of original side interfaces morphology in Fig. 5(c–f). The oxide scale thickness formed on the bottom is about 50 nm. The diffusion of Cr, Co, Ni, and Re elements seems week in depth direction.

**Figure 10:**
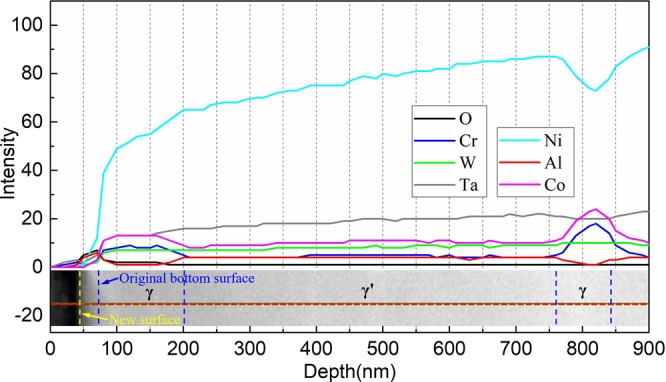
EDS line scans in the depth direction of specimen after oxidation experiment.

Figure 10 shows the element distribution crossed both γ and γ′ phases in the depth direction, the scanning line is shown as the red dashed line in Fig. 9(b). It can be seen clearly that the diffusion happened only at the area closest to the surface. There is a quite thin layer oxidation laying on the interface of γ/γ′ phase, it corresponds exactly to the severely oxidized region in Fig. 9(a) and its value is about 30 nm.

**Figure 11:**
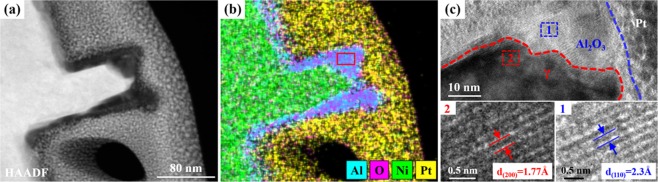
Composition and structure of oxide scale. (**a**) HAADF-STEM image of oxide scale and superalloy. (**b**) EDS mapping of oxide scale and superalloy. (**c**) High-resolution STEM images of oxide scale and superalloy.

To be more accurate in analyzing the formed oxide scale, a detail TEM experiment was carried out. Figure 11(a) is the HAADF-STEM image of oxide scale and superalloy, it should be noted that the other elements including Cr, Co and Re are in the matrix superalloy instead of oxide scale, those elements are not shown in Fig. 11(b). The ratio of O and Al (the zone marked in red box of Fig. 11(b)) counted by EDS is 1.44, which is extremely close to the elemental ratio of Al_2_O_3_. Besides, the high-resolution STEM images (Fig. 11c) further supports the findings. The crystal plane spacing of blue dashed box zone marked in Fig. 11c is calculated and the value is 2.3 Å, which corresponds with the [110] crystal plane spacing of α-Al_2_O_3_^22^. The crystal plane spacing of red dashed box zone is 1.77 Å, corresponding with the [200] crystal plane spacing of Ni (fcc crystal structure).

## Discussion

### Oxide scale mechanism

**Figure 12:**
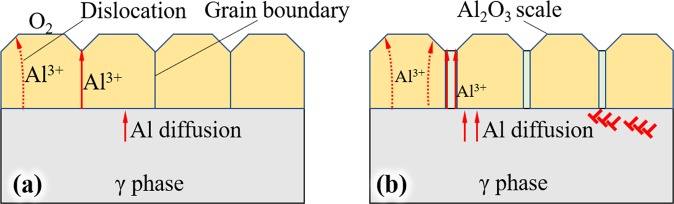
Oxidation mechanism of γ phase in single crystal superalloy. (**a**) only thermal exposure, (**b**) thermal exposure and stress loaded.

After admitting oxygen, O_2_ molecules dissociate and diffuse on the surface of γ phases, that process also reported on the surface of Cu-Au alloy^23^. Compared with Ni elements, Al shows greater affinity for O, then Al atoms diffuse toward the alloy-oxygen interface, and may also transport through cation vacancy^24^. Once Al_2_O_3_ precipitates nucleated on the original surface of γ phase as shown in Fig. 4(b–d). Al ion can transport by dislocations in oxide scale and boundaries between Al_2_O_3_ grains, the scale grows in form of outward transport^25^, such a process is shown in Fig. 12(a).

When stress is loaded, the dislocation in γ phase will increase and gather in the interface of γ and γ′ phases, which leads to the increase of vacancy^26,27^. The vacancies and channels formed by dislocation will enhance the diffusion of Al atoms, as illustrated by Fig. 12(b). Also, the dislocation in Al_2_O_3_ grain and defects on grain boundaries in oxide scale is likely to increase during loading stress, the Al ions transport along those defects will enhance too, which caused the growth of oxide scale showed an accelerating trend, as evident from Fig. 5(a–f).

### Oxide scale kinetics

**Figure 13:**
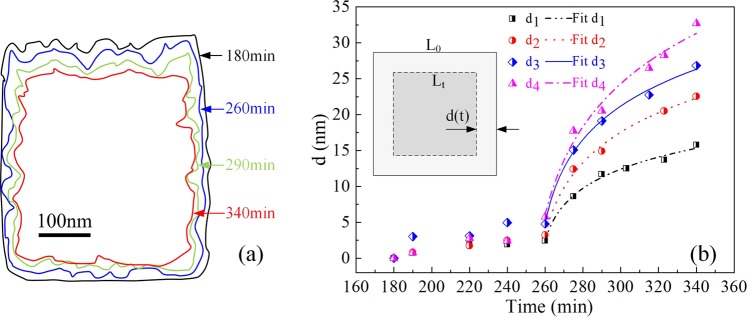
(**a**) Surrounding areas by oxide, (**b**) thickness of oxide at different times.

To quantify the degree of oxidation, the oxide-oxygen interface was outlined using red dashed lines shown as Fig. 4(a). In this paper, four zones were selected, changes in each area were measured by Software Image J at different times, as outlined in Fig. 13(a). In thermal exposure period, slightly moving is founded, shown as the black outline and blue outline in Fig. 13(a). Once stress is loaded, the growth of oxide becomes considerable, displayed as green and red outline in Fig. 13(a). Since the shape is almost square, the slightly zigzag of outlines could be ignored compared with the whole shape. Therefore, the thickness of oxide at different times could be described as

$${\rm{d}}({\rm{t}})=\frac{{S}_{{\rm{t}}}-{S}_{0}}{({L}_{0}+{L}_{{\rm{t}}})/2}=\frac{2({S}_{{\rm{t}}}-{S}_{0})}{\sqrt{{S}_{0}}+\sqrt{{S}_{{\rm{t}}}}}$$

Where d(t) is the thickness of oxide, *S*_t_ and *S*_0_ are the area of surrounded by oxide at t moment and initial time. *L*_t_ and *L*_0_ are the side length of the surrounding area at t moment and initial time, respectively.

The thickness of oxide in sideway direction at different times is shown as scatter graph in Fig. 13(b). At early stage of initial oxidation, oxidation of metal proceeds at a constant rate, which obeys the “linear rate law”, this period is well verified especially for the thickness of area 3 increasing during 180 min to 260 min. In that stage, chemisorption of oxygen is the rate-controlling step studied by Pettit^28^ and has been described by Neil^29^. However, in this paper, the rate during thermal exposure period is quite small, since the probability of oxygen molecules colliding with surface of alloy is low caused by high vacuum in SEM chamber. Once stress is loaded, the oxidation rate shows exceptional increase possibly triggered by diffusion enhancement of Al atom or ion in alloy and oxide scale. Moreover, logarithmic model (d(t) = A + *k*_ln_(t + B)) still fits well with the oxidation process during thermal exposure with stress loaded, which indicates the stress doesn’t make the oxide scale deform to a large extent, and diffusion is still the rate-controlling step.

The value of fitting parameters for the four areas were compared in Table 1. It can be appreciated that the value of *k*_ln_ increase with the area increasing, which means the larger area or the longer side appears more oxidized phenomenon. This is mainly caused by higher density of dislocation in longer side of γ phase, and the high density of dislocation enhancing the diffusion process of Al atoms.

**Table 1:** Oxidation rate constants during thermal exposure and stress loaded period.

area	A	*k*_ln_	B
1	−5.0	4.6	−255.0
2	−12.4	7.8	−252.4
3	−10.5	8.3	−253.6
4	−23.9	12.2	−248.5

## Conclusions

Oxide nucleation and growth on a nickel-based single crystal superalloy during only temperature of 1150 °C and thermal-stress pattern (1150 °C and /330 MPa) was observed. The oxide scale grown on the interface of γ/γ′ phase was constituted of α-Al_2_O_3_ precipitates. Loading stress enhanced the diffusion of Al atom in γ phase and Al ion in the oxide scale and caused high oxidation rate. Logarithmic model fitted well with the oxidation process during thermal exposure with stress loaded
